# Comparison of Aerosol Generation Between Bag Valve and Chest Compression-Synchronized Ventilation During Simulated Cardiopulmonary Resuscitation

**DOI:** 10.3390/jcm14196790

**Published:** 2025-09-25

**Authors:** Young Taeck Oh, Choung Ah Lee, Daun Choi, Hang A. Park

**Affiliations:** 1 Department of Emergency Medicine, Hallym University College of Medicine, Dongtan Sacred Heart Hospital, Hwaseong-si 18450, Republic of Korea; bluethin@cau.ac.kr (Y.T.O.);; 2 Hallym Dongtan Simulation Center, Hwaseong-si 18450, Republic of Korea

**Keywords:** aerosol-generating ventilation, cardiopulmonary resuscitation, aerosols, mechanical ventilation, manual ventilation, positive-pressure respiration, Bayesian analysis

## Abstract

**Background**: Cardiopulmonary resuscitation can generate aerosols, potentially exposing healthcare workers (HCWs) to infection. Bag valve ventilation (BV) is widely used but is prone to aerosol dispersion, whereas chest compression-synchronized ventilation (CCSV) maintains a closed respiratory circuit. In this study, we compared aerosol generation between CCSV and BV during chest compressions following endotracheal intubation in a simulated resuscitation setting. **Methods**: In a randomized crossover design, 12 sessions each of CCSV and BV were conducted on an intubated manikin undergoing mechanical chest compressions for 10 min. Aerosols with ≤5-μm diameter were generated using a saline nebulizer and measured every minute with a particle counter positioned 50 cm from the chest compression site. Bayesian linear regression of minute-by-minute log-transformed aerosol particle counts was used to estimate group differences, yielding posterior means, 95% credible intervals, and posterior probabilities. **Results**: The aerosol particle counts increased during the initial 3 min with the use of both methods. Thereafter, the aerosol particle counts with CCSV stabilized, whereas those with BV continued to increase. From 4 to 10 min, the posterior probability that CCSV generated fewer particles exceeded 0.98, peaking at 9 min. Both peak and time-averaged log-transformed aerosol particle counts were significantly lower with CCSV than with BV (*p* = 0.010 and *p* = 0.020, respectively). **Conclusions**: In this simulation, CCSV generated significantly fewer aerosols than BV did during chest compressions, with differences emerging after 4 min and persisting thereafter. Thus, CCSV may reduce aerosol exposure of HCWs, supporting its early implementation during resuscitation in infectious disease settings.

## 1. Introduction

Cardiopulmonary resuscitation (CPR) is a lifesaving intervention for cardiac arrest; however, it poses a significant risk of aerosol generation and potential infection transmission to healthcare workers (HCWs), particularly during respiratory pandemics, such as coronavirus disease 2019 [1,2]. Airway management procedures performed during CPR have been recognized as potential sources of infectious airborne particles [3,4]. Therefore, both research and clinical guidelines have emphasized the need for enhanced infection control strategies during CPR to protect HCWs from diseases transmitted through aerosols and droplets [5,6].

Manual ventilation using bag valve mask (BVM) has been associated with substantial dispersion of exhaled air and aerosol particles, potentially increasing the risk of pathogen transmission to HCWs [7]. Although recent evidence suggests that endotracheal intubation may not generate as much aerosol as previously believed, leading to its removal from the infection prevention and control hazard lists [8], the need for early and controlled airway management during resuscitation remains critical. Current resuscitation guidelines emphasize balancing high-quality CPR while implementing strategies to minimize infection risk for HCWs. These recommendations emphasize the need to identify ventilation approaches that not only ensure effective oxygen delivery but also reduce aerosol exposure during CPR.

There are several options for providing ventilation after endotracheal intubation during CPR. Bag valve ventilation (BV) is the most widely used method for providing ventilation during CPR. Another option is mechanical ventilation. Chest compression-synchronized ventilation (CCSV), developed by Kill et al. [9], has demonstrated improved arterial oxygenation during CPR when used in conjunction with mechanical chest compression devices [10]. CCSV may limit aerosol dispersion by preserving a closed respiratory circuit and minimizing disconnections [11,12].

Although both simulation and clinical studies have examined aerosol generation with various ventilation methods [2,13], direct quantitative comparisons between BV and CCSV during chest compressions remain limited. This gap highlights the need for further research to guide infection control strategies during CPR. Given the heightened attention to infection prevention and the evolving recommendations for respiratory support in high-risk environments [14,15], robust experimental data are needed to inform clinical practice. Therefore, in this simulation study, we aimed to quantitatively compare aerosol generation between BV and CCSV during chest compressions using an intubated manikin, employing real-time particle measurements, to inform future infection control strategies.

## 2. Materials and Methods

### 2.1. Experimental Design

**Figure 1:**
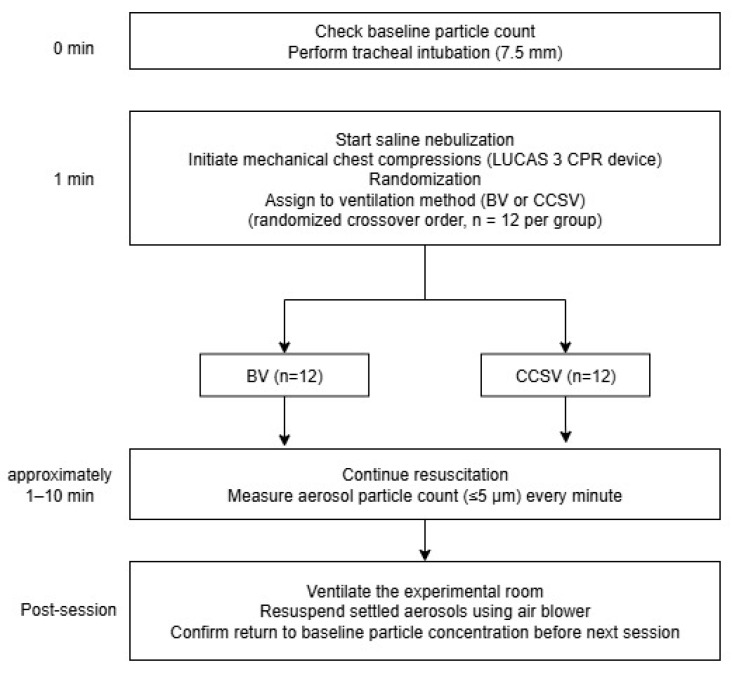
Study protocol diagram. Abbreviations: CPR, cardiopulmonary resuscitation; BV, manual bag valve ventilation; CCSV, chest compression-synchronized ventilation.

This was a manikin-based simulation study, conducted using a randomized crossover design (Figure 1). A Resusci Anne First Aid Full Body TrolleySuit (Laerdal, Stavanger, Norway) was modified with the Resusci Anne Airway Head Upgrade Kit to enable both chest compressions and endotracheal intubation. Before each trial, the manikin was intubated with a 7.5 mm endotracheal tube. Mechanical chest compressions were continuously delivered using a LUCAS 3 CPR device (Stryker, Kalamazoo, MI, USA) for 10 min per trial, at a rate of 102 ± 2/min and a depth of 53 ± 2 mm. These settings were consistent with the default device settings and international resuscitation guidelines. Manual BVs were performed using a self-inflating resuscitation bag (Ambu^®^ SPUR^®^ II, adult size, Ambu A/S, Copenhagen, Denmark) equipped with a pressure-limiting valve and a resuscitator volume of approximately 1547 mL. BVs were delivered approximately every 6 s, corresponding to a 10:1 compression-to-ventilation ratio. Each breath was delivered over approximately 1 s, to achieve visible chest wall expansion of the manikin without excessive airway pressure. To prevent hyperventilation, only one-third of the bag volume was compressed for each breath. All BVs were performed by individuals who were trained to follow a standardized protocol, ensuring consistency within and across resuscitation sessions. For CCSV, a MEDUMAT Standard^2^ ventilator (Weinmann Emergency Medical Technology GmbH + Co. KG, Hamburg, Germany) was used. Ventilator parameters were set to a peak inspiratory pressure of 60 cm H_2_O, inspiratory time of 205 ms, and fraction of inspired oxygen of 1.0. The synchronization algorithm of CCSV inherently minimized conflicts between compressions and ventilations, whereas manual BV was performed during ongoing compressions to reflect common clinical practice and avoid interruptions in perfusion. Importantly, no high-efficiency particulate air (HEPA) filters were used in either the BV or ventilator circuits, as the simulations were designed to model a worst-case scenario and directly compare aerosol generation between the two methods without the confounding effect of filtration.

The sample size was determined from a pilot simulation study. The crossover design allowed paired comparisons between the two ventilation methods within each simulation session. The pilot study yielded a mean log-transformed paired difference of 0.13 with a standard deviation (SD) of the paired differences of 0.04 (Cohen’s *d_p_* approximately = 3.25). Based on this effect size, a paired *t*-test would require three pairs (three sessions, each testing both BV and CCSV) to achieve 80% power at a two-sided significance level of 0.05. However, to account for potential experimental variability and in line with pilot study recommendations of a minimum of 12 trials per group [16,17], in the main study, we conducted 12 sessions of each ventilation method. This exceeded the minimum required sample size, ensuring adequate statistical power. The order of ventilation methods was randomized to minimize potential carryover effects inherent in crossover designs.

All experiments were conducted in a negative-pressure isolation room to minimize environmental contamination and facilitate efficient removal of airborne particles. Negative pressure was maintained at −12.4 Pa, with room temperature set at 25 °C and relative humidity set between 50% and 55%. No external airflow disturbances occurred during the experiments, and ambient lighting and ventilation conditions were held constant across all sessions.

### 2.2. Aerosol Generation and Particle Measurement

We generated aerosols using a Sileo 54 nebulizer system (Macjin Medical, Seoul, Korea) using saline. The device operates at a maximum compressor pressure of 282.6 kPa (±10%) and a maximum flow rate of 9 L/min (±10%), producing aerosol particles with a mass median aerodynamic diameter ≤5.0 μm. For the experiment, the nebulizer was connected to the manikin’s lung to release aerosols into its airway.

**Figure 2:**
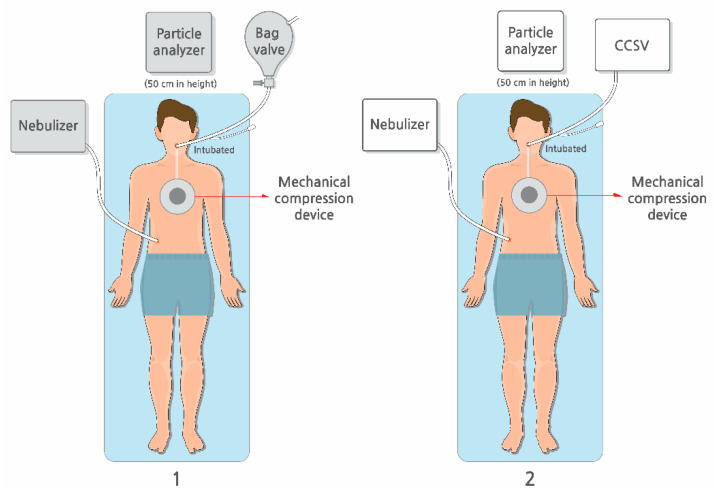
Study setting. The experiment was conducted, comparing (1) manual bag valve ventilation with (2) mechanical ventilation using CCSV during chest compressions conducted using the LUCAS3 CPR device. Abbreviations: CCSV, chest compression-synchronized ventilation; CPR, cardiopulmonary resuscitation.

Particle concentrations were recorded at 1 min interval using a PMD 331 particle counter (Temtop, Elitech Technology, San Jose, CA, USA) positioned 50 cm from the chest compression site to approximate the location of the rescuers (Figure 2). The majority of particles detected measured 0.3 μm in diameter, indicating that most aerosols generated were submicron in diameter. After each session, the negative-pressure ventilation system operated for at least 10 min, and the next trial was started only after baseline particle concentrations returned to pre-experiment levels.

### 2.3. Statistical Analysis

For descriptive and trend analyses, the mean and SD values for aerosol particle counts (<5 μm in diameter) were calculated for each ventilation method and time point, with 95% confidence intervals (CIs). These data were used to generate time-series line plots with shaded 95% CI bands to illustrate temporal trends in aerosol generation for each ventilation method.

Bayesian linear regression was performed independently for each time point using log-transformed aerosol particle counts as the dependent variable and ventilation method as the independent variable, assuming a Gaussian likelihood. Weakly informative priors were specified (Normal [0, 100,000] for the intercept and group effect; Cauchy [0, 10,000] for residual variance). For each time point, posterior means and 95% credible intervals of the group difference were estimated, along with the posterior probability that CCSV generated lower aerosol particle counts than BV did (P [CCSV < BV]).

All analyses were generated in R (version 4.3.3) using the tidyverse (version 2.0.0) and brms (version 2.22.0) packages. Statistical significance was defined as a 95% credible interval excluding zero or a posterior probability ≥0.975 that CCSV generated lower aerosol particle counts than BV did.

## 3. Results

**Figure 3:**
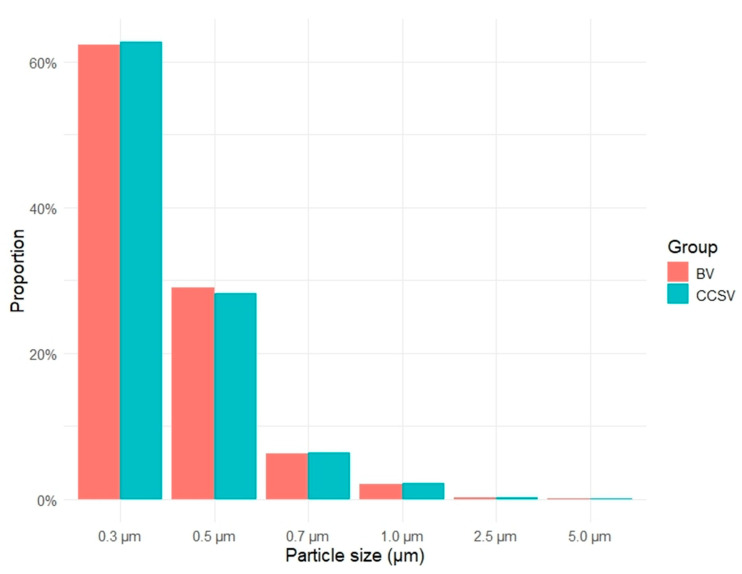
Proportion of detected aerosol particle sizes for each ventilation method. Abbreviations: BV, manual bag valve ventilation; CCSV, chest compression-synchronized ventilation.

The aerosol particle size distribution for both ventilation methods is presented in Figure 3. In each group, particles measuring 0.3 μm in diameter predominated, accounting for more than 60% of the total aerosol particle count, followed by particles of 0.5 μm and 0.7 μm diameter. The overall particle size distributions were similar between the groups.

**Figure 4:**
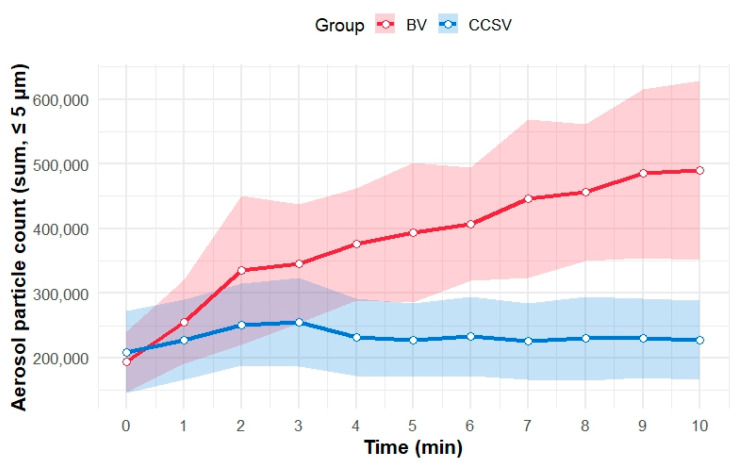
Temporal trends in aerosol particles (≤5 μm in diameter) during simulated CPR for the two ventilation methods. The mean aerosol particle counts (sum of particles ≤ 5 μm in diameter) are shown as solid lines for each minute for both methods: BV (red) and CCSV (blue). Shaded areas represent the 95% confidence intervals for the mean value at each time point. Abbreviations: BV, manual bag valve ventilation; CCSV, chest compression-synchronized ventilation; CPR, cardiopulmonary resuscitation.

Figure 4 presents the temporal trends in mean aerosol concentrations of particles <5 μm in diameter for each ventilation method. From the start of the experiment to 3 min, the mean aerosol particle counts increased in both groups. Thereafter, the aerosol particle counts with CCSV plateaued, whereas those with BV continued to increase. After 5 min, the 95% CIs for the two ventilation methods no longer overlapped.

Table 1 summarizes the Bayesian posterior estimates of the differences in aerosol particle counts (CCSV-BV) for particles (<5 μm in diameter) at each minute during the simulation. At baseline (minute 0), the aerosol particle counts were comparable between the groups (mean difference [CCSV-BV]: 13,450; 95% credible interval: −61,661 to 88,936; posterior probability [CCSV < BV]: 0.35). After 4 min, the mean differences in the aerosol particle counts were consistently negative, and the 95% credible intervals excluded zero, indicating lower counts in the CCSV group than in the BV group. The posterior probability that CCSV produced fewer aerosols than BV did exceeded 0.98 for 4–10 min, peaking at 9 min (mean difference = −156,903 particles; 95% credible interval: −277,002 to −24,118).

**Figure 5:**
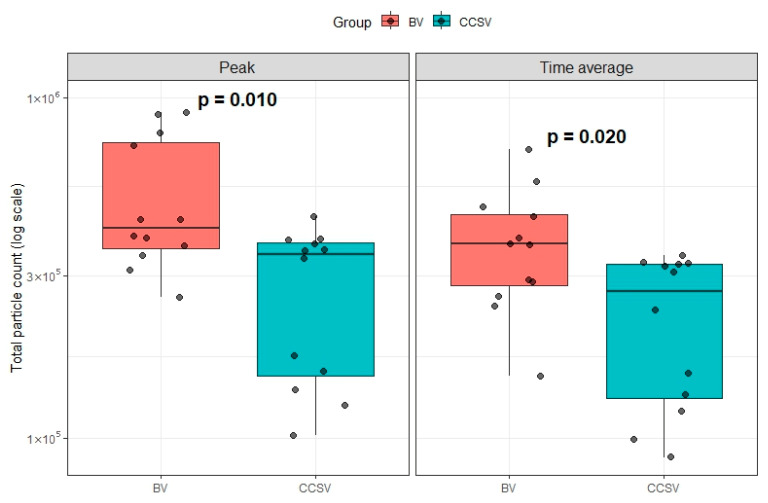
Comparison of peak and time-averaged aerosol particle counts for BV and CCSV. Boxes indicate the interquartile range, horizontal lines within boxes represent the median, and whiskers show the range of values. Abbreviations: BV, manual bag valve ventilation; CCSV, chest compression-synchronized ventilation.

Furthermore, on the log scale, both peak aerosol particle counts (*p* = 0.010) and log-scaled time-averaged aerosol particle counts (*p* = 0.020) were significantly lower in the CCSV group than in the BV group (Figure 5).

## 4. Discussion

This randomized crossover simulation study quantitatively compared aerosol generation between CCSV and BV during mechanical chest compressions using a manikin. The mean aerosol particle counts increased with both ventilation methods during the first 4 min. However, a clear divergence emerged thereafter: CCSV consistently produced fewer aerosols than BV did, and this difference persisted through the 10 min simulation period. The Bayesian posterior estimates further supported this finding. From 4 min onward, the posterior probability that CCSV generated fewer aerosols than BV did exceeded 0.98, indicating that, given our data, there is greater than a 98% probability that CCSV produces fewer aerosols than BV does after 4 min of CPR. This suggests that CCSV is likely to reduce aerosol exposure, compared with BV, after the initial minutes of resuscitation, highlighting its potential relevance in clinical settings. Furthermore, both the peak and time-averaged aerosol particle counts were significantly lower in the CCSV group than in the BV group. These findings suggest that CCSV may be more advantageous than BV in reducing aerosol exposure during CPR and that the difference becomes more pronounced over time, highlighting the importance of selecting the ventilation method early in resuscitation.

Chan et al. reported substantial aerosol dispersion during manual ventilation with a BVM in a manikin model [7]. In their laser-visualization experiments, dispersion was strongly influenced by tidal volume and mask seal integrity, highlighting the potential infection risk associated with ventilation using BVM during CPR. Although manual ventilation was associated with higher aerosol generation in both Chan et al.’s study and ours, a key methodological difference is that Chan et al. evaluated manual ventilation before intubation using BVM, when mask leakage can be a major contributor to aerosol spread, whereas our study examined manual BV delivered via an endotracheal tube after intubation, eliminating mask-fit variables.

Our findings are consistent with recent evidence indicating that tracheal intubation, when performed under controlled conditions, may not be a high-risk aerosol-generating procedure [18]. Dhillon et al. reported that endotracheal intubation in an operating room did not markedly increase aerosol particle counts compared with baseline and produced substantially less aerosol than coughing did. Similarly, Hung et al. found that both intubation and extubation generated aerosol levels well below those generated by coughing [19]. In contrast, facial mask ventilation, especially with an imperfect seal, was associated with greater aerosol generation. This supports the rationale that early intubation may help control aerosol exposure during resuscitation by eliminating mask leakage and allowing for more controlled ventilation.

Our study showed that manual BV produced higher aerosol particle counts than CCSV did, even after endotracheal intubation. The difference became significant over time, with both peak and average aerosol particle counts being consistently lower in the CCSV group versus the BV group. These findings provide quantitative support for the hypothesis that closed-circuit ventilation, such as CCSV, can reduce infectious aerosol exposure, compared with manual ventilation, even in intubated patients. This aligns with the findings of Avari et al., who demonstrated that closed-loop systems equipped with high-efficiency particulate air filters substantially reduced environmental contamination, compared with open-circuit systems, such as non-invasive bilevel positive-pressure ventilation, non-rebreather face masks, and high-flow nasal oxygen [20]. Therefore, it is important to apply closed-circuit ventilation early in resuscitation to minimize aerosol exposure.

This study specifically compared a closed-circuit system using CCSV with BV in the intubated state. Although other closed-circuit approaches, such as intermittent positive pressure ventilation using standard mechanical ventilators, may produce different outcomes, they were not included in this analysis due to important clinical limitations. Conventional ventilation modes are not tailored for cardiac arrest scenarios as they deliver breaths at fixed intervals, independent of chest compressions. Consequently, chest compressions can inadvertently trigger ventilator breaths, potentially leading to hyperventilation and elevated airway pressures. These risks limit the use of standard mechanical ventilators during CPR, and current resuscitation guidelines continue to recommend manual ventilation in such cases. In contrast, CCSV is specifically designed for use during CPR. It detects each chest compression and delivers a breath in synchrony with the rise in airway pressure generated during the compression phase [21]. This synchronization is thought to help maintain lung volume, minimize air leakage, and enhance cardiac output by optimizing thoracic pressure dynamics. Additionally, CCSV may reduce the risk of progressive atelectasis by avoiding cyclical alveolar recruitment and derecruitment [10]. These physiological advantages highlight the potential of CCSV as a practical and physiologically tailored closed-circuit ventilation strategy for use during CPR, compared with other modes.

Evidence from both clinical and experimental studies indicates that chest compressions or defibrillation followed by chest compressions can generate aerosols, with documented cases of infection transmission to HCWs during resuscitation [2,22]. This underscores the need for a precautionary approach, including appropriate personal protective equipment, to protect HCWs from potentially life-threatening infections [23]. Although controlled studies have shown that tracheal intubation is a non-aerosol-generating procedure and carries less risk than ventilation using BVM does, rescuers remain positioned near the patient’s head during ventilation and therefore are at a potential risk. Few studies have directly compared closed-loop ventilation systems with manual BV in intubated patients. Demonstrating reduced aerosol generation with closed-loop systems is important for ensuring HCW safety during CPR.

This study has a few limitations. First, as this was a simulation study conducted in an isolation room using a saline nebulizer to simulate aerosols, it may not fully reflect real clinical conditions. Patient-derived aerosols, which often contain biological secretions, may differ in particle size distribution, composition, and dispersion behavior, compared with those produced by a saline nebulizer. On one hand, the absence of patient secretions and the inability to reproduce sudden bursts of aerosols that may occur during chest compressions or defibrillation could have led to an underestimation of aerosol dispersion. On the other hand, the nebulizer continuously produced fine submicron particles that may differ from patient-derived aerosols in terms of the size distribution and settling behavior, potentially resulting in an overestimation of dispersion. Moreover, real patients may present a wide range of physiological variability, including airway resistance, lung compliance, secretion burden, and dynamic responses to ventilation, all of which could substantially influence aerosol behavior. In addition, human factors, such as inconsistent bagging technique, mask seal quality, provider fatigue, and procedural timing, can affect ventilation quality and aerosol escape in clinical settings. To mitigate this, we standardized environmental parameters and applied a crossover design. Because our primary aim was to compare the differences between BV and CCSV rather than quantify absolute aerosol particle counts, the trend and direction of change over time are more meaningful than the exact particle counts. Second, as the simulation employed a manikin and a mechanical chest compression device, the results may differ from those in real patients and when performed manually by HCWs. However, to reduce variability, CPR guidelines were strictly followed to ensure that compressions were delivered consistently. Given these limitations, the findings of this study are intended to provide conceptual insights into infection control strategies and highlight the potential benefits of closed-circuit ventilation approaches compared with manual BV. These results should not be interpreted as a substitute for clinical guidelines. Further clinical studies in real-world patient populations are necessary to validate these results before they can be incorporated into evidence-based recommendations. Finally, although our sample size exceeded the minimum requirement derived from a pilot study, the relatively small number of simulation sessions remains a limitation. Variability in aerosol generation, due to environmental fluctuations, device performance, or operator technique, may have influenced the precision of our estimates and effective statistical power. Although the crossover design helped mitigate some of this variability, larger studies are needed to validate the robustness and generalizability of these findings. Future research should also compare various closed-circuit ventilation strategies during CPR, evaluate their impact on aerosol generation, and determine the optimal balance between infection control and ventilation effectiveness.

## 5. Conclusions

In this crossover simulation study, CCSV generated significantly fewer aerosols than BV during chest compressions, with differences becoming apparent after 4 min and persisting thereafter. These findings suggest that closed-circuit ventilation strategies may reduce HCWs’ aerosol exposure during resuscitation. Early implementation of such systems should be considered; however, owing to inherent limitations of simulation studies, the results should be interpreted as supportive evidence to inform infection control strategies. Further clinical research is needed to confirm these findings before integrating CCSV into CPR protocols.

## Figures and Tables

**Table 1:** Bayesian posterior estimates at each minute for differences in aerosol particle counts between CCSV and BV during simulation.

Minute	Mean Difference (CCSV-BV)	95% Credible Interval	Posterior P [CCSV < BV]
0	13,450	−61,661 to 88,936	0.35
1	−22,577	−106,822 to 62,011	0.71
2	−56,209	−163,690 to 58,298	0.85
3	−66,380	−165,283 to 33,996	0.90
4	−109,307	−204,614 to −12,465	0.99
5	−117,119	−223,643 to −5939	0.98
6	−129,865	−227,683 to −25,963	0.99
7	−144,158	−257,197 to −21,335	0.99
8	−155,785	−266,242 to −33,859	0.99
9	−156,903	−277,002 to −24,118	0.99
10	−149,564	−279,073 to −9282	0.98

Abbreviations: BV, manual bag valve ventilation; CCSV, chest compression-synchronized ventilation.

## Data Availability

The data presented in this study are available from the corresponding author upon reasonable request.

## Abbreviations

The following abbreviations are used in this manuscript:

CPR	Cardiopulmonary resuscitation
HCWs	Healthcare workers
BV	Bag valve ventilation
CCSV	Chest compression-synchronized ventilation
BVM	Bag valve mask
SD	Standard deviation
CI	Confidence interval
